# Nanofiltration Membranes Formed through Interfacial Polymerization Involving Cycloalkane Amine Monomer and Trimesoyl Chloride Showing Some Tolerance to Chlorine during Dye Desalination

**DOI:** 10.3390/membranes12030333

**Published:** 2022-03-17

**Authors:** Micah Belle Marie Yap Ang, Yi-Ling Wu, Min-Yi Chu, Ping-Han Wu, Yu-Hsuan Chiao, Jeremiah C. Millare, Shu-Hsien Huang, Hui-An Tsai, Kueir-Rarn Lee

**Affiliations:** 1 R&D Center for Membrane Technology and Department of Chemical Engineering, Chung Yuan Christian University, Taoyuan 32023, Taiwan; yiling890510@gmail.com (Y.-L.W.); chuminyi1029@gmail.com (M.-Y.C.); pinghanwu@cycu.org.tw (P.-H.W.); msdonaldc@hotmail.com (Y.-H.C.); huian@cycu.edu.tw (H.-A.T.); 2 Research Center for Membrane and Film Technology, Department of Chemical Science and Engineering, Kobe University, Rokkodaicho 1-1, Nada, Kobe 657-8501, Japan; 3 School of Chemical, Biological, and Materials Engineering and Sciences, Mapúa University, Manila 1002, Philippines; jcmillare@mapua.edu.ph; 4 Department of Chemical and Materials Engineering, National Ilan University, Yilan 26047, Taiwan; 5 Research Center for Circular Economy, Chung Yuan Christian University, Taoyuan 32023, Taiwan

**Keywords:** cycloalkane amine, dye desalination, chlorine-resistant membrane, nanofiltration, polyamide membrane

## Abstract

Wastewater effluents containing high concentrations of dyes are highly toxic to the environment and aquatic organisms. Recycle and reuse of both water and dye in textile industries can save energy and costs. Thus, new materials are being explored to fabricate highly efficient nanofiltration membranes for fulfilling industrial needs. In this work, three diamines, 1,4-cyclohexanediamine (CHD), ethylenediamine (EDA), and p-phenylenediamine (PPD), are reacted with TMC separately to fabricate a thin film composite polyamide membrane for dye desalination. Their chemical structures are different, with the difference located in the middle of two terminal amines. The surface morphology, roughness, and thickness of the polyamide layer are dependent on the reactivity of the diamines with TMC. EDA has a short linear alkane chain, which can easily react with TMC, forming a very dense selective layer. CHD has a cyclohexane ring, making it more sterically hindered than EDA. As such, CHD’s reaction with TMC is slower than EDA’s, leading to a thinner polyamide layer. PPD has a benzene ring, which should make it the most sterically hindered structure; however, its benzene ring has a pi-pi interaction with TMC that can facilitate a faster reaction between PPD and TMC, leading to a thicker polyamide layer. Among the TFC membranes, TFC_CHD_ exhibited the highest separation efficiency (pure water flux = 192.13 ± 7.11 L∙m^−2^∙h^−1^, dye rejection = 99.92 ± 0.10%, and NaCl rejection = 15.46 ± 1.68% at 6 bar and 1000 ppm salt or 50 ppm of dye solution). After exposure at 12,000 ppm∙h of active chlorine, the flux of TFC_CHD_ was enhanced with maintained high dye rejection. Therefore, the TFC_CHD_ membrane has a potential application for dye desalination process.

## 1. Introduction

The disposal of highly contaminated wastewater containing dyes can be toxic to both land-based and aquatic organisms. Synthetic dyes are used in many industries, especially the textile, leather, paper, food, and packaging industries. A huge amount of wastewater is regularly generated, which requires special attention for its treatment [1,2]. An example of a synthetic dye is brilliant blue R, which has been intensively used in clinical and biochemical laboratories for the staining and assessment of proteins. It is highly toxic and non-biodegradable because it is highly resistant to heat, light, and acidic conditions. Therefore, removal of brilliant blue R before the disposal of wastewater into the environment is essential in order to prevent environmental pollution that could affect human health [3,4,5,6,7].

To optimize the separation process, recycling, and reuse of the valuable chemical components (dyes) and water from the wastewater, several different unit operations have been proposed. A combination of physical, chemical, biological, and/or membrane treatments have been explored to process the waste streams, which contain huge amounts of dyes [8]. The inefficiency of conventional treatment systems opens the door for membrane separation processes. Among the pressure-driven membrane processes, nanofiltration is frequently chosen because of its relatively higher flux than reverse osmosis and relatively higher selectivity than ultrafiltration membranes [9].

At present, thin-film composite (TFC) nanofiltration membranes are still preferred commercially to treat effluents containing dyes [9]. They are commonly prepared through interfacial polymerization of diamines with polyacyl chloride, and consequently, a selective polyamide layer can be formed on top of a porous support. However, the efficiency and chlorine-resistance of the polyamide membrane requires improvement to keep up with the pace of industrialization and to match the supply and demand. Because bleaching is also performed to remove color from the fabrics, some chlorine and other impurities might be present in the dye wastewater [10]. Furthermore, chlorine also helps to remove the foulants and natural organic matter from the membrane surface [11,12]. Hence, different strategies and techniques have been employed to fabricate a highly efficient nanofiltration membrane with high chlorine resistance.

Strategies such as varying the monomer properties [13,14,15,16], embedding nanofillers [17,18,19], tailoring of the membrane support properties [20,21], inclusion of gutter layers [22,23], and surface modification [24,25,26,27,28] are widely explored to improve separation efficiency and chemical resistance. For example, Feng et al. [29], used 3,5-diamino-1,2,4-triazol as an amine monomer, and reacted it with TMC to make a polyamide membrane for dye/salt separation. They found that this N-rich amine monomer can provide better chlorine resistance than commercial membranes. Yu et al. [30] reacted m-phenylenediamine-4-methyl and cyclohexane-1,3,5-tricarbonyl chloride to fabricate a polyamide membrane. The used of acyl chloride with a cyclohexane structure reduced the amount of benzene on the membrane surface, resulting in an improved chlorine resistance. Liu et al. [31] used two monomers in an aqueous phase, m-xylylenediamine and polyethyleneimine. They found that the addition of m-xylylenediamine improved the chlorine resistance of the NF membrane. Because of the existence of -CH_2_ between the amine and the benzene ring in m-xylylenediamine, the chance of chlorine degradation was reduced. Exploring new potential diamine monomers is a direct strategy to achieve the possibility of gaining a higher separation efficiency. Aromatic monomers, such as m-phenylenediamine, have been commonly used in interfacial polymerization. However, in diamine monomers with aromatic rings, their aromatic rings can undergo direct chlorination, which will deteriorate the polyamide layer with long-term exposure to chlorine [32]. Hence, diamines with alkanes have been explored for their potential as an alternative monomer for nanofiltration membranes with high chlorine resistance.

In this work, three diamine monomers, CHD, EDA, and PPD, were explored for use in the fabrication of TFC membranes with chlorine resistant properties. CHD and EDA have an alkane chemical structure with diamine terminal groups, whereas PPD has a benzene ring with a diamine as a para-substituent. The physicochemical properties, separation efficiency, and chlorine resistance of the TFC membranes are correlated and discussed. This study may potentially contribute to the practical application of nanofiltration membranes in different industry sectors.

## 2. Materials and Methods

### 2.1. Materials

PAN powder, used without purification, was given by Tong-Hwa Synthesis Fiber Co. Ltd. (Taipei, Taiwan). The monomers for interfacial polymerization were as follows: p-phenylenediamine, C_6_H_4_ (NH_2_)_2_ (PPD, Alfa Aesar, Heysham, Lacashire, UK); ethylenediamine, C_2_H_4_(NH_2_)_2_ (EDA, Alfa Aesar, Heysham, Lacashire, UK); 1,4-cyclohexanediamine, C_6_H_14_N_2_ (CHD, Tokyo Chemical Industry Co. Ltd., Tokyo, Japan); and trimesoyl chloride, C_9_H_3_Cl_3_O (TMC, Tokyo Chemical Industry Co. Ltd., Tokyo, Japan). N-methyl-2-pyrrolidone and n-Hexane were solvents of PAN and TMC, respectively, and were procured from Tedia Company Inc. (Fairfield, OH, USA). Salts (MgCl_2_, MgSO_4_, Na_2_SO_4_, and NaCl) were purchased from Sigma-Aldrich (Saint Louis, MO, USA). Two commercial membranes (NF90 and NF270) were bought from DuPont, Taiwan. Brilliant blue R, as dye, was provided by Tokyo Chemical Industry Co. Ltd. (Tokyo, Japan). The distilled water was laboratory-produced using a Lotun Technic machine (Lotun Technic Co. Ltd., New Taipei, Taiwan).

### 2.2. Fabrication of Thin-Film Composite Membranes

A total of 600 g of PAN powder was dissolved in 3400 mL NMP inside an agitator mixer at 60 °C for 1 day, where the concentration of PAN was 15 wt%. Then it was transferred to a 5 L bottle and degassed overnight. Afterwards, the solution was poured into a continuous casting machine (casting knife gap = 200 µm) installed with a non-woven polyester with water as a coagulation bath. PAN precipitated rapidly on the surface of the non-woven polyester upon contact with water. A roll of PAN support was prepared and was placed in another machine to remove excess NMP by washing it with water.

**Figure 1:**
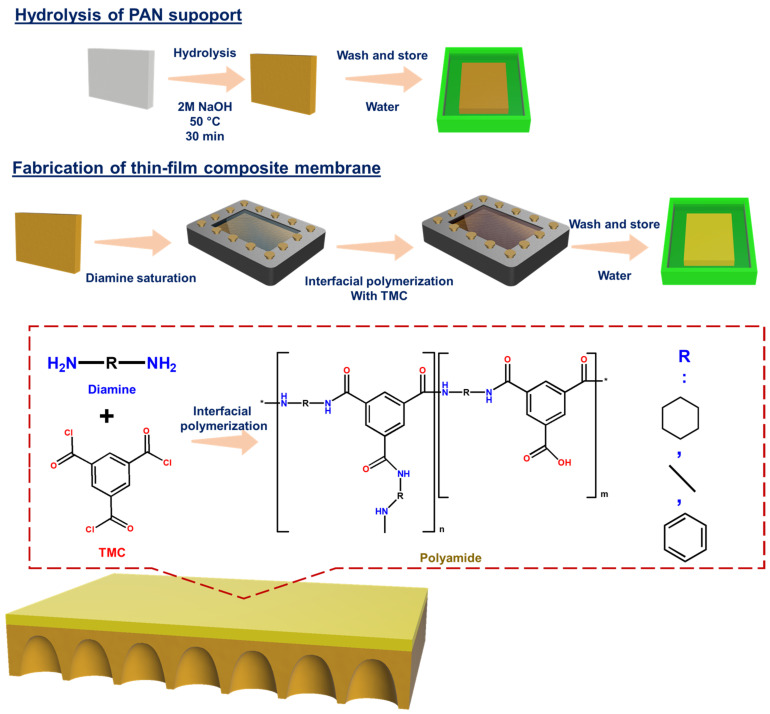
Schematic diagram of the membrane preparation.

The PAN support was cut to 19 × 19 cm before it underwent hydrolysis and was utilized for interfacial polymerization (Figure 1). The cut outs were soaked in a 2M NaOH solution at 50 °C for 30 min. Afterwards, it was washed with distilled water until the pH of the distilled water reached 7. These were then stored in distilled water prior to interfacial polymerization. The hydrolyzed PAN support is denoted as HPAN.

The wet HPAN support was clamped onto a stainless-steel plate. Subsequently, a 100 mL aqueous diamine solution (0.35 wt%) was poured on top of it. After 2 min, the solution was removed and any excess droplets on top of the membrane were removed using an air gun. Then, the TMC/n-hexane solution (0.2 wt%) was poured on top, and a thin polyamide layer immediately formed. The reaction of diamine and TMC took place for 1 min. Afterwards, it was transferred to distilled water to wash off excess reactants and was later stored in distilled water prior to filtration testing. For characterization, the membranes were dried using a vacuum before utilizing.

### 2.3. Membrane Characterization

Surface chemical properties were analyzed using attenuated total reflectance-Fourier transform infrared (ATR-FTIR) spectroscopy (Perkin Elmer Spectrum 100 FTIR Spectrometer, Waltham, MA, USA) and X-ray photoelectron spectroscopy (XPS, VG K-alpha ThermoFisher Scientific, Inc. Waltham, MA, USA). Surface morphology and cross-sectional images were captured using field emission scanning electron microscopy (FESEM, S-4800, Hitachi Co, Tokyo, Japan). Atomic force microscopy (AFM, NanoScope^®^ V, Bruker, Billerica, MA, USA) mapped the surface morphology to quantify the surface roughness (root mean square, Rq) of the membranes. The water contact angle of the membranes was measured using an automatic interfacial tensiometer (PD-VP Model, Kyowa Interface Science Co. Ltd., Niiza-City, Saitama, Japan). The surface charges of the membranes at pH 3, 7, and 11 were determined using SurPASS Electrokinetic Analyzer (Anton Paar, NSW, Australia).

### 2.4. Filtration Test

Four pieces of membranes were placed separately in the membrane cell of the lab-made crossflow filtration setup. All membranes underwent pre-compaction at 6.5 bar for 1 h. Afterwards, the pure water flux (*J*) was measured at 6 bar by collecting the total mass of the water in permeate (*m*) over a certain time period (*t*). Salt rejection was determined by feeding 1000 ppm of salts. The same membrane was used to determine the dye rejection, but the membranes were washed for 1 h by feeding distilled water to ensure no excess salts were trapped in the membrane. Then, the membrane cells containing the membranes were transferred to a similar setup that was used only for dye filtration. Aqueous brilliant blue R solution (50 ppm) was fed to the filtration set-up. After 10 min, the permeate was collected to determine the dye rejection using ultraviolet-visible spectroscopy (UV/Vis) (BioTek Instruments Inc., Winooski, VT, USA). Flux and dye rejection were calculated using Equations (1) and (2):

(1) $$J=\frac{m}{\rho At}$$

(2) $$R=\frac{C_{f}-C_{i}}{C_{f}}\times 100\%$$

where *A* was the effective area of the membrane (12.57 cm^2^), ρ was the water density (1 kg/L), and *C_f_* and *C_i_* were the concentrations of solutes in the feed and permeate, respectively. The concentration of salts was determined using a conductivity meter, Mettler Toledo SevenMulti (Schwerzenbach, Switzerland). A standard curve was plotted for the conductivity vs. the concentration of salts. From this standard curve, the concentration of permeate was determined.

### 2.5. Evaluation of Chlorine Resistance

Membranes were immersed in a 2000 ppm NaOCl solution for 0.5 to 6 h. Afterwards, it was rinsed and stored in distilled water. The flux and the dye rejection were examined using the same procedure as in Section 2.4. Commercial membranes, such as NF90 and NF270, were also used to compare the membrane performance after exposure to a NaOCl solution.

## 3. Results and Discussion

### 3.1. Surface Chemical Property

**Figure 2:**
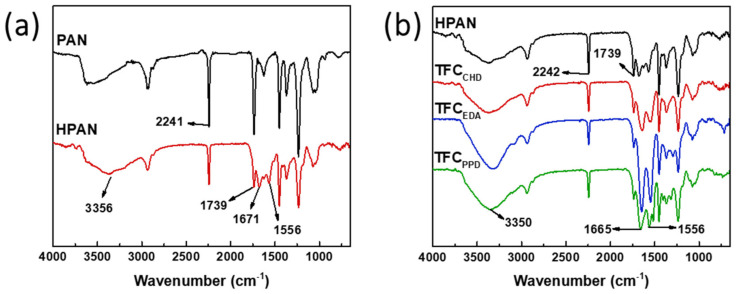
(**a**) ATR-FTIR spectra of the PAN, HPAN, and (**b**) TFC membranes.

Figure 2 shows the ATR-FTIR spectra of the PAN, HPAN and TFC membranes. PAN had a characteristic peak at 2241 cm^−1^, indicating the stretching vibration of CN bands. The peak at 1739 cm^−1^ was from the presence of CO stretching bands. After alkaline treatment using NaOH, the peak of HPAN at 2242 cm^−1^ was decreased, indicating the successful hydrolysis of PAN [33,34]. After interfacial polymerization, the intensity of the peaks of TFC membranes at 1665 and 1556 cm^−1^ was increased, indicating the presence of primary amide (amide I, C=O) and secondary amide (amide II, N-H), respectively [35]. TFC_EDA_ had the highest intensity of amide I, suggesting that it had the highest degree if cross-linking. Furthermore, from the XPS analysis (Table 1), the N/O had the following order: TFC_EDA_ > TFC_PPD_ > TFC_CHD_. A higher N/O ratio means that the crosslinking degree is higher, implying that TFC_EDA_ had the highest crosslinking degree.

### 3.2. Morphology and Roughness

**Figure 3:**
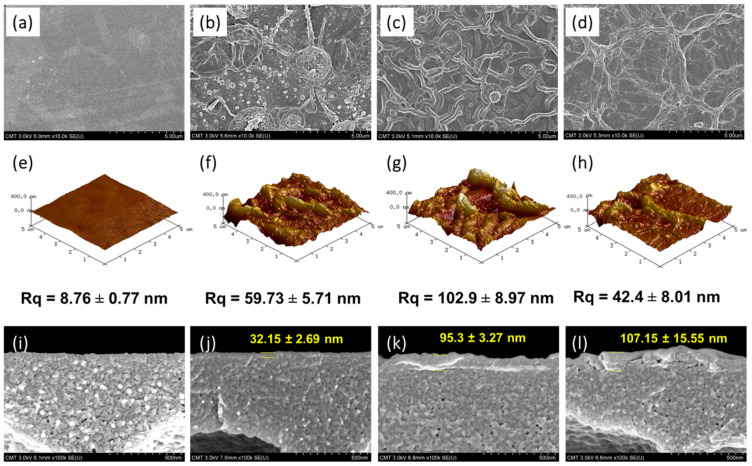
Surface and cross-sectional FESEM images, and surface roughness of (**a**,**e**,**i**) HPAN, (**b**,**f**,**j**) TFC_CHD_, (**c**,**g**,**k**) TFC_EDA_, and (**d**,**h**,**l**) TFC_PPD_.

The surface roughness was measured using AFM analysis. HPAN had a smooth surface Figure 3a,e of Rq = 8.76 ± 0.77 nm. A dissimilar surface morphology was observed for the TFC membranes when using different diamines (Figure 3b–d). The surface roughness exhibited a decreasing order as follows: TFC_EDA_ (Rq = 102.9 ± 8.97 nm) > TFC_CHD_ (Rq = 59.73 ± 5.71 nm) > TFC_PPD_ (Rq = 42.4 ± 8.01 nm). EDA and CHD had an alkane structure, but EDA had a short linear structure, where both amine terminals of EDA can readily react with acyl chloride in TMC. The cyclohexane of CHD had a more sterically hindered structure, which slowed the reaction with TMC. Thus, EDA reacted faster with TMC than CHD. Hence, TFC_EDA_ (95.3 ± 3.27 nm) had a relatively thicker polyamide layer than that of TFC_CHD_ (32.15 ± 2.69 nm) and had the highest degree of crosslinking (Figure 2 and Table 1). The benzene ring in PPD had caused it to be the most sterically hindered structure, but its pi-pi interaction between PPD and TMC could have facilitated an increase in reactivity, which provided a thicker polyamide layer (107.15 ± 15.55 nm). Compared with the commercial membrane surface (Figure S1), the developed membrane also contained some ridge structures and had uniform structure throughout the surface.

### 3.3. Surface Hydrophilicity and Charged

**Figure 4:**
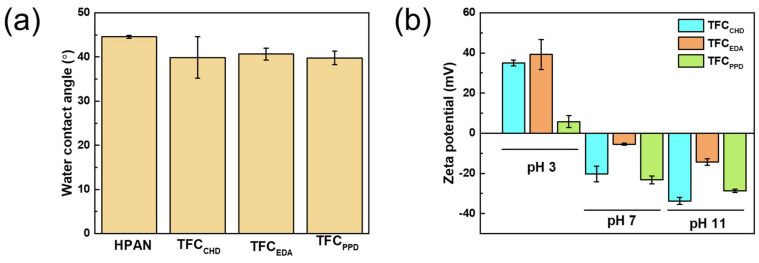
(**a**) Water contact angle at 1 min and (**b**) surface zeta potential of TFC membranes.

Figure 4a presents the water contact angle of HPAN and TFC membranes at 1 min. HPAN had a water contact angle of 44.55 ± 0.35°, which is similar to our previous work [36]. After interfacial polymerization of diamine and TMC on the surface of the HPAN support, the water contact angle of the TFC membranes was 39–40°. Hydrophilicity of the membranes was affected by the surface roughness or surface functional groups. Generally, a hydrophilic surface with a rougher surface had a lower contact angle than that of a smoother surface [37]. In these results, TFC_EDA_ had the roughest surface, but its degree of crosslinking was the highest, producing low hydrophilic functional groups on the surface. Therefore, the water contact angle was similar for all TFC membranes because the surface roughness and surface functional groups were compromising on each other.

Figure 4b shows the surface charges of the membranes at different pH levels. Polyamide membrane had an amphoteric surface, where the acidic group was the COOH from hydrolysis of TMC, and the basic groups were either -NH_2_ or =NH. This implied that the surface charge density of the membrane could vary depending on the pH of the solution [38]. At pH 3, all TFC membranes had a positively charged surface, because at lower pH, the protonation of the amine groups occurred. However, at pH 7 and 11 the TFC membranes had a negatively charged surface, suggesting the dissociation of the carboxylic acid groups [39]. Herein, the TFC_EDA_ membrane had a highly positively charged density at pH 3, but less negatively charged at pH 7 and 11, meaning that it had a high amount of crosslinked amide groups or excess amines formed on the membrane surface. This was evident in the ATR-FTIR and XPS results (Figure 1 and Table 1), where the TFC_EDA_ had the highest peak intensity of the amide I group. On the other hand, the TFC_PPD_ had the most negatively charged surface, indicating that there was more carboxylic acid dissociated on the membrane surface at pH 7. The pi-pi interaction of the PPD and TMC induced faster reactivity between PPD and TMC, leading to a denser initial layer formed in the polyamide near the membrane surface. However, when they continued to react for a longer period, the PPD would no longer transfer to the interface during interfacial polymerization, because the first dense polyamide layer prevented it. When this happens, there is not enough PPD to react with TMC, therefore, the unreacted acyl chloride of TMC would hydrolyze and provide more COOH groups on the surface. TFC_CHD_ was the most negatively charged at pH 11, indicating that most of its COOH group on the surface had dissociated to COO-, because it had the lowest ratio of N/O on the surface (Table 1) that led to the high negative charge of the membrane. However, at pH 3, it had higher zeta potential than that of TFC_PPD_ because TFC_CHD_ had more amides or free amines on the surface than TFC_PPD_. Therefore, at pH 7, the high amount of amines in TFC_CHD_ neutralized some of the carboxyl groups on the surface, emitting less negative charge than TFC_PPD_.

### 3.4. Membrane Separation Performance and Chlorine Resistant Test

**Figure 5:**
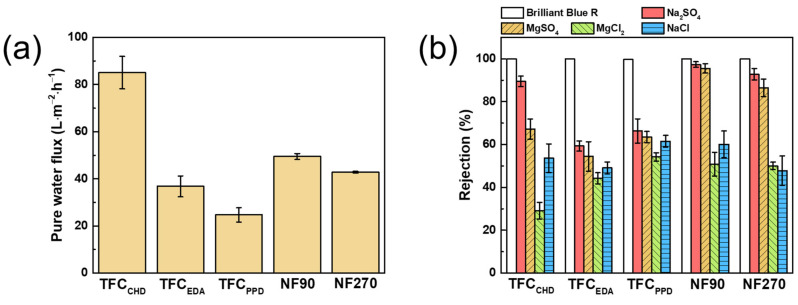
(**a**) pure water flux and (**b**) salt and dye rejection of the TFC and commercial membranes.

Figure 5 demonstrates the separation efficiency of the TFC membranes. TFC_CHD_ had the highest water permeability, whereas TFC_PPD_ had the lowest water permeability. This was consistent with the average thickness of the polyamide layer formed, where TFC_CHD_ had the thinnest and TFC_PPD_ had the thickest polyamide layer. The thicker the polyamide layer, the greater the mass transfer resistance of water, thus giving a lower water permeability. Moreover, TFC_EDA_ had lower water permeability than that of TFC_CHD_ because of the reaction between EDA and TMC produces a polyamide layer with a higher degree of crosslinking (Table 1). However, the salt rejection for TFC_EDA_ was also lower than that of TFC_CHD_, meaning that the polyamide produced by EDA and TMC was not dense enough to separate salt. Furthermore, compared with TFC_CHD_, the zeta potential of TFC_EDA_ at pH 7 was less negatively charged, resulting in lower rejection of divalent ions. All TFC membranes had more than 99% rejection of the dye. Furthermore, all membranes displayed typical salt rejection for a membrane with a negatively charged surface at pH 7: Na_2_SO_4_ > MgSO_4_ > NaCl > MgCl_2_ [40]. Compared with the commercial membranes (NF90 and NF270), TFC_CHD_ had a higher water permeability and dye/salt selectivity. Thus, in the following section, the concentration for CHD and TMC were chosen to be optimized for further study because TFC_CHD_ provide the highest separation efficiency.

**Figure 6:**
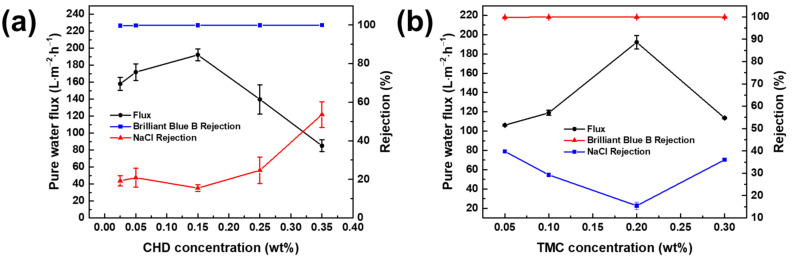
Effect of (**a**) CHD concentration and (**b**) TMC concentration on the performance of the TFC_CHD_ membrane.

Figure 6a reveals the effect of CHD concentration on the performance of the TFC_CHD_ membrane. The concentration of TMC was fixed at 0.2 wt% and the contact time for the aqueous phase and reaction time with TMC/n-hexane solution were 2 min and 1 min, respectively. When raising the concentration of CHD from 0.025 to 0.15 wt%, the pure water flux also increased from 157.95 ± 7.45 to 192.13 ± 7.11 L∙m^−2^∙h^−1^. At a low concentration of the amine, there was just enough CHD that can react with the acyl chloride group of TMC, meaning that the hydrolysis of the acyl chloride groups of TMC was less likely to occur. If less hydrolysis of TMC occurred, there were less linear groups on the polyamide layer and more cross-linked groups, giving a more compact polyamide layer and resulting in lower water flux. From 0.15 to 0.35 wt% CHD, the flux declined from 192.13 ± 7.11 to 85.03 ± 6.89 L∙m^−2^∙h^−1^, as at high concentrations of amine, more amines could react with TMC, producing a thicker and denser polyamide layer. Therefore, 0.15 wt% CHD was the optimal concentration.

Figure 6b illustrates the effect of TMC concentration on the performance of the TFC_CHD_ membrane at a fixed concentration of CHD (0.15 wt%) The contact time for the aqueous phase and reaction time for the TMC/n-hexane solution were 2 min and 1 min, respectively. The optimal concentration of TMC was 0.2 wt%, where the performance of TFC_CHD_ was as follows: pure water flux = 192.13 ± 7.11 L∙m^−2^∙h^−1^, dye rejection = 99.92 ± 0.10%, NaCl rejection = 15.46 ± 1.68%. There was a trade-off between the water flux and the NaCl rejection, suggesting that the TFC_CHD_ had the loosest structure at a TMC concentration of 0.2 wt%. At a low concentration of TMC, there were enough acyl chloride groups that could react with CHD, where the hydrolysis of the acyl chloride group of TMC were less likely to occur, thus providing a higher degree of crosslinking, resulting in a lower flux. On the other hand, a high concentration of TMC could also result in a high degree of crosslinking and a denser polyamide layer. A similar trend was also observed in our previous work [41].

**Figure 7:**
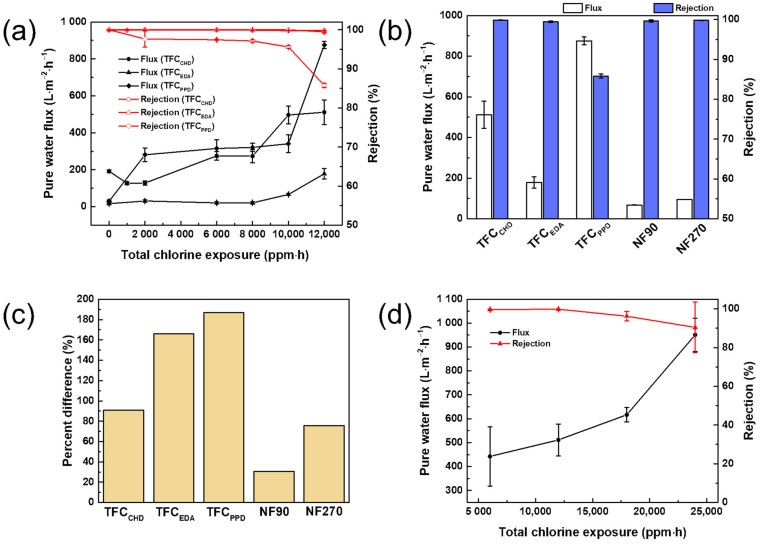
(**a**) Performance of TFC membranes at different chlorine exposure and (**b**) comparison of the chlorine resistances of the TFC membranes in this work with commercial membranes (total chlorine exposure = 12,000 ppm∙h). (**c**) Percent difference before and after chlorine exposure of the membranes (total chlorine exposure = 12,000 ppm∙h). (**d**) Effect of chlorine concentration (exposure time = 6 h) Amine monomer concentration = 0.15 wt%; TMC concentration = 0.2 wt%.

During the water treatment process, injection of chlorine to treat the liquid waste was implied. However, at a certain dosage of chlorine, the polyamide layer could deteriorate [42,43,44]. Thus, a membrane with high tolerance for chlorine was fabricated for practical use. Figure 7a,b show the performance of TFC membranes after exposure to a 2000 ppm NaOCl solution from 0 to 6 h. The flux of TFC_CHD_ decreased from 0 to 2000 ppm∙h, as the active chlorine diminished the hydrogen bonding between the polyamide chains. This led to a movement of the polymer chain that would result to compaction of the polymer chain under pressure [45]. At a longer exposure time of the TFC_CHD_ to the active chlorine, the pure water flux increased to 511.36 ± 66.57 L∙m^−2^∙h^−1^, where the amide bonds underwent hydrolysis, resulting in a looser polyamide structure [46]. Nevertheless, the dye rejection of TFC_CHD_ remained at more than 99%. For TFC_EDA_, the flux maintained similarly from 0 to 8000 ppm∙h; after exposing it to 10,000 to 12,000 ppm∙h, its flux increased to 66.34 ± 5.52 to 178.38 ± 27.58 L∙m^−2^∙h^−1^, implying that the active chlorine weakened the polyamide bonding in TFC_EDA_ at the said dosage, but the dye rejection remained unaffected. At a low dosage of the chlorine, TFC_PPD_ started to decline its dye rejection. After exposure in active chlorine at 12,000 ppm∙h, its dye rejection declined to 85.79%, with an increase of pure water flux up to 875.28 ± 19.05 L∙m^−2^∙h^−1^ because TFC_PPD_ had more benzene rings that were susceptible to chlorine attack. The benzene ring had undergone irreversible direct aromatic chlorination, leading to deterioration of the polyamide layer. The overall results also implied that exposure to active chlorine can be used as a post-treatment to increase the separation efficiency of the membrane with the dye. Among the membranes, TFC_CHD_ had the highest separation efficiency. Comparing the TFC membranes in this work from two commercial membranes, NF90 and NF270, the TFC_CHD_ membrane had the highest separation efficiency, suggesting the superior properties of the membrane. After exposure in chlorine, the flux for NF90 was increased from 49.49 ± 1.24 to 67.33 ± 0.96 L∙m^−2^∙h^−1^, whereas the flux of NF270 was increased from 42.80 ± 0.38 to 94.92 ± 0.47 L∙m^−2^∙h^−1^. Both NF90 and NF270 had a rejection for brilliant blue R of over 99%. Figure 7c presents the percent difference of the pure water flux before and after exposure in chorine at 12,000 ppm∙h. Both commercial membranes had a low percent difference. TFC_PPD_ had the highest percent difference because it is susceptible to a chlorine attack due to the presence of many benzenes ring on its structure. Even if the TFC_EDA_ and TFC_CHD_ membranes had high percentage differences before and after exposure in chlorine, they still provided acceptable performances for dye desalination processes. Figure 7d plots the effect of chlorine concentration vs. the flux and rejection of TFC_CHD_. From 1000 to 4000 ppm, the flux increased from 442.47 ± 124.05 to 950.75 ± 70.19 L∙m^−2^∙h^−1^, whereas the dye rejection also declined from 99 to 90%. This indicated that the TFC_CHD_ can maintain over 90% rejection at very high concentrations of chlorine.

## 4. Conclusions

Choosing a suitable monomer for fabricating a highly chlorine-resistant polyamide nanofiltration membrane is important. Different chemical structures of diamines have different reaction rates with TMC, which can affect the surface morphology, roughness, polyamide thickness, hydrophilicity, and charge density. A suitable chemical structure can provide a high separation efficiency with high chlorine resistance. TFC_CHD_ prepared with cycloalkane diamines had the slowest reaction rate of studied membranes, because it has a strong steric hindrance, resulting in a thinner polyamide layer and the lowest degree of crosslinking. No difference in water contact angle was found. Thus, the membrane thickness, crosslinking, and surface charges were responsible for the separation efficiency of the membrane. Both before and after chlorine treatment, TFC_CHD_ still exhibited the highest separation efficiency, with a water flux from 192.13 ± 7.11 to 511.36 ± 66.57 L∙m^−2^∙h^−1^, while maintaining over 99% dye rejection. Furthermore, it has less benzene rings in the polyamide amide layer, making it more resistant to active chlorine. Therefore, post-treatment using chlorine to enhance the performance of TFC_CHD_ can also be applied to obtain a desirable membrane performance.

## Figures and Tables

**Table 1:** Elemental analysis of TFC membranes using XPS.

	C	O	N	N/O
TFC_CHD_	75.39	16.7	7.91	0.473653
TFC_EDA_	67.71	18.96	13.33	0.703059
TFC_PPD_	71.12	19.24	9.65	0.501559

## Data Availability

The data presented in this study are available on request from the corresponding author.

## Supplementary Materials

The following supporting information can be downloaded at: https://www.mdpi.com/article/10.3390/membranes12030333/s1, Figure S1: FESEM images of commercial membranes.
