# Supplementary material for: Nanofiltration Membranes Formed through Interfacial Polymerization Involving Cycloalkane Amine Monomer and Trimesoyl Chloride Showing Some Tolerance to Chlorine during Dye Desalination
Source: Membranes (Basel). 2022 Mar 17;12(3):333. doi: 10.3390/membranes12030333 (PMC8954597; doi:10.3390/membranes12030333)
Supplement: Supplementary file 1 [file membranes-12-00333-s001.zip › membranes-1556659-supplementary.pdf]

membranes

Supplementary Material for

Nanofiltration Membranes Formed through Interfacial Polymerization Involving Cycloalkane Amine Monomer and Trimesoyl Chloride Showing Some Tolerance to Chlorine during Dye Desalination

Micah Belle Marie Yap Ang [1,*], Yi-Ling Wu [1], Min-Yi Chu [1], Ping-Han Wu [1], Yu-Hsuan Chiao [1,2], Jeremiah C. Millare [3], Shu-Hsien Huang [1,4,*], Hui-An Tsai [1] and Kueir-Rarn Lee [1,5,*]

1 R&D Center for Membrane Technology and Department of Chemical Engineering, Chung Yuan Christian University, Taoyuan 32023, Taiwan; yiling890510@gmail.com (Y.-L.W.); chuminyi1029@gmail.com (M.-Y.C.); pinghanwu@cycu.org.tw (P.-H.W.); huian@cycu.edu.tw (H.-A.T.)
2 Research Center for Membrane and Film Technology, Department of Chemical Science and Engineering, Kobe University, Rokkodaicho 1-1, Nada, Kobe 657-8501, Japan; msdonaldc@hotmail.com (Y.-H.C.);
3 School of Chemical, Biological, and Materials Engineering and Sciences, Mapúa University, Manila 1002, Philippines; jcmillare@mapua.edu.ph (J.C.M.)
4 Department of Chemical and Materials Engineering, National Ilan University, Yilan 26047, Taiwan
5 Research Center for Circular Economy, Chung Yuan Christian University, Taoyuan 32023, Taiwan
* Correspondence: mbmyang@gmail.com (M.B.M.Y.A.); huangsh@niu.edu.tw (S.-H.H.); krlee@cycu.edu.tw (K.-R.L.)

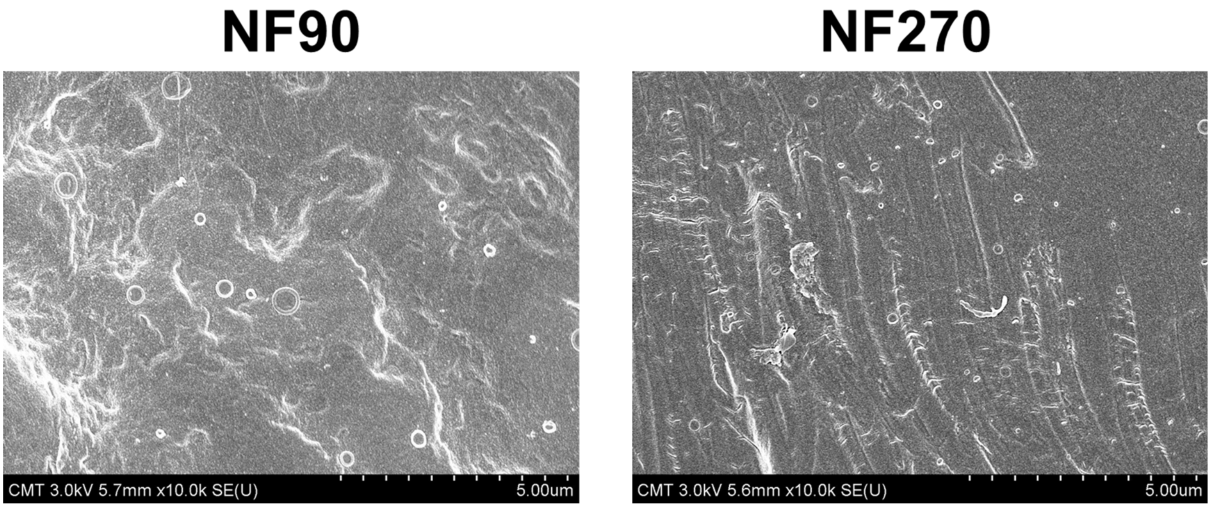

Figure S1. FESEM images of commercial membranes.

Adsorption study:

The adsorption study was followed from this work [1], but modified by contacting only the surface of the membrane with the dye solution. Briefly, wet membranes was clamp in a self-made vessel. Afterwards, 50 mL of 50 ppm brilliant blue R was poured into the vessel. The vessel was placed in the shaker at 100 rpm. The concentration of the dye in the vessel was measured at time t, using ultraviolet-visible spectroscopy, UV/Vis, Bio-Tek Instruments Inc., Winooski, USA.

1. B.M. Thamer, A. Aldalbahi, M. Moydeen A, H. El-Hamshary, A.M. Al-Enizi, M.H. El-Newehy, Effective adsorption of Coomassie brilliant blue dye using poly(phenylene diamine)grafted electrospun carbon nanofibers as a novel adsorbent, Mater. Chem. Phys. 234 (2019) 133-145. https://doi.org/10.1016/j.matchemphys.2019.05.087.
